# Supplementary material for: Detection of microplastics in the feline placenta and fetus
Source: PLoS One. 2025 Apr 2;20(4):e0320694. doi: 10.1371/journal.pone.0320694 (PMC11964201; doi:10.1371/journal.pone.0320694)
Supplement: Attachment — Submitted filename: Response to reviewers.docx [file pone.0320694.s001.docx]

# Reviewer Questions and Responses

| Question | Response |
| --- | --- |
| The authors should clarify why a single foetus with his placenta was included from each pregnancy and what criterion was used to select it (always the same position within the uterus?). | The separation of the placenta from the endometrium during an early stage of pregnancy is particularly challenging due to the fragility of the tissues, as well as the uneven number and distribution of placentas. To ensure uniformity in the samples, it was decided to use only one fetus for which complete separation of the placenta was successfully achieved. Line 150-156 |
| Foetal age should be reported for the 8 pregnancies, notwithstanding the fact that no gestation age-related condition can be tested when only 8 pregnancies are included. | Foetal ages for the 8 pregnancies have been reported as follows: 15, 20, 27, 27, 28, 29, 30, and 30 days, with a mean age of 26.0 days. (Line 110-111) |
| I wonder how an intracardiac injection can be done in 15-day embryos/foetuses (see Knospe (2002) Periods and Stages of the Prenatal Development of the Domestic Cat). | Thank you for your important observation. The injections were performed in the thoracic region in the bigger fetuses; however, given the extremely small size of the fetuses and the inability to confirm the actual distribution of the drug within the circulatory system, it is more accurate to refer to these as intracoelomic injections. We have updated the phrasing accordingly. |
| Is it true that a coloured particle for which the polymer matrix cannot be identified should be more correctly identified as a ‘suspect’ MNP. If so, this should be reported, modifying the results from line 261 to 271, Page 15. | We thank the referee for this important comment. We agree that the colored particles for which we were unable to identify the polymer matrix should be classified as suspect MNPs, and we have revised the discussion accordingly. Furthermore, we have moved the discussion about the Raman spectra of dyes/polymer matrix to this paragraph. |
| I would add some comments on the possibility of contamination (see your Reference n 32 on human placentae collected at C-sections). | Thank you for your comment. Throughout all stages of processing, plastic instruments were never used. The laboratory environments were controlled. Digestion procedures were performed under a fume hood. It has been reported that microplastics are present in the air within various indoor environments (Zhang et al., 2020). To address this concern, uteri were carefully washed with sterile saline solution prior to dissection to minimize the risk of external contamination. We added few lines in the discussion section. Lines 315-320 |
| On page 15 (line 261) I do not understand where number 5 comes from: four pregnancies were contaminated by coloured MNPs (cats 4,5,6 and 7). In two cases (cat 4 foetus and cat 5 placenta) PE was detected. I cannot see 5. | The number of analysed sample is 16 (8 placentas and 8 fetuses). Out of these, we found 5 contaminated samples, belonging to 4 animals (in one case we found that both placenta and fetus were contaminated). In the text, we specified that the 5 samples belong to 4 animals. |
| On page 16 (lines 290-291), there is a wrong reference number and a wrong citation: if the correct number is 13 and not 31 (please, add brackets), tap water resulted contaminated with higher levels of MNPs than bottled water. | Thank you, corrected. |
| I would stress in the conclusions the alarming findings of this work, that should be a stimulus to drastically limit/eliminate the use of plastic and, for scientists, to find valid alternatives to it. | Thank you for your suggestion. Added at line 311-315. |
| Page 4, line 58: ‘(MPs’ delete ‘(‘ | Done |
| Page 7 line 121-122: Propofol is not part of premedication, but it is used for anaesthesia induction. | Thank you for your comment. Corrected. Line 142-143 |
| Page 12, line 212: ‘is’ instead of ‘in’. | Corrected |
| Line 294: do the two ranges refer to the heart? It is not clear. | The typo was corrected |
| Table 1: The columns do not correspond to what is indicated in the legend. | Corrected. In the legend, columns 2 and 3 were mistakenly swapped. |
| Why two corresponding authors?! It is rather unusual. | Given that the work was carried out by researchers from different areas of expertise (clinical and spectroscopic laboratory), it was decided to designate two corresponding authors, each representing their respective field of expertise. |
